# Lipid Emulsion Enhances Vasoconstriction Induced by Dexmedetomidine in the Isolated Endothelium-Intact Aorta

**DOI:** 10.3390/ijms22073309

**Published:** 2021-03-24

**Authors:** Soo Hee Lee, Seong-Ho Ok, Seung Hyun Ahn, Hyun-Jin Kim, Sung Il Bae, Ji-Yoon Kim, Kyeong-Eon Park, Yeran Hwang, Ju-Tae Sohn

**Affiliations:** 1 Department of Anesthesiology and Pain Medicine, Gyeongsang National University Hospital, 15 Jinju-daero 816 Beon-gil, Jinju-si 52727, Gyeongsangnam-do, Korea; lishiuji@naver.com (S.H.L.); ash3832@gmail.com (S.H.A.); snugsoul@naver.com (S.I.B.); avadore33@naver.com (J.-Y.K.); kep1574@naver.com (K.-E.P.); babi9876@naver.com (Y.H.); 2 Department of Anesthesiology and Pain Medicine, Gyeongsang National University Changwon Hospital, 11 Samjeongja-ro, Seongsan-gu, Changwon-si 51472, Gyeongsangnam-do, Korea; mdoksh@naver.com; 3 Department of Anesthesiology and Pain Medicine, Gyeongsang National University College of Medicine, 15 Jinju-daero 816 Beon-gil, Jinju-si 52727, Gyeongsangnam-do, Korea; mdoksh@naver; 4 Institute of Health Sciences, Gyeongsang National University, Jinju-si 52727, Korea; 5 Korea Division of Applied Life Sciences (BK21 Plus), Gyeongsang National University, Gyeongsang 52828, Korea; hyunjkim@gnu.ac.kr; 6 Department of Food Science & Technology, Institute of Agriculture and Life Science, Gyeongsang National University, Gyeongsang 52828, Korea; 7 Department of Anesthesiology and Pain Medicine, Gyeongsang National University College of Medicine, Gyeongsang National University Hospital, 15 Jinju-daero, 816 Beon-gil, Jinju-si 52727, Gyeongsangnam-do, Korea

**Keywords:** dexmedetomidine, lipid emulsion, nitric oxide, contraction, endothelial nitric oxide synthase

## Abstract

This study aimed to examine the effect of lipid emulsion (LE) on the vasoconstriction induced by dexmedetomidine (DMT) in the isolated rat aorta and elucidate the associated cellular mechanism. The effect of LE, N^W^-nitro-L-arginine methyl ester (L-NAME), and methyl-β-cyclodextrin (MβCD) on the DMT-induced contraction was examined. We investigated the effect of LE on the DMT-induced cyclic guanosine monophosphate (cGMP) formation and DMT concentration. The effect of DMT, LE, 4-Amino-3-(4-chlorophenyl)-1-(t-butyl)-1H-pyrazolo[3,4-d]pyrimidine,4-Amino-5-(4-chlorophenyl)-7-(t-butyl)pyrazolo[3,4-d]pyrimidine (PP2), and rauwolscine on the phosphorylation of endothelial nitric oxide synthase (eNOS), caveolin-1, and Src kinase was examined in the human umbilical vein endothelial cells. L-NAME, MβCD, and LE (1%, standardized mean difference (SMD): 2.517) increased the DMT-induced contraction in the endothelium-intact rat aorta. LE (1%) decreased the DMT (10^−6^ M) concentration (SMD: −6.795) and DMT-induced cGMP formation (SMD: −2.132). LE (1%) reversed the DMT-induced eNOS (Ser1177 and Thr496) phosphorylation. PP2 inhibited caveolin-1 and eNOS phosphorylation induced by DMT. DMT increased the Src kinase phosphorylation. Thus, LE (1%) enhanced the DMT-induced contraction by inhibition of NO synthesis, which may be caused by the decreased DMT concentration. DMT-induced NO synthesis may be caused by the increased eNOS (Ser1177) phosphorylation and decreased eNOS (Thr495) phosphorylation potentially mediated by Src kinase-induced caveolin-1 phosphorylation.

## 1. Introduction

Dexmedetomidine (DMT), which is a highly selective alpha-2 adrenoceptor agonist (α_2_:α_1_ = 1620:1), is widely used for sedation [1,2]. A small dose of DMT (0.2 μg/kg/h and 0.6 μg/kg/h) causes a slightly decreased mean arterial blood pressure, whereas a high dose of DMT (1.25 μg/kg and 2.5 μg/kg) causes an initial increase in mean arterial pressure, followed by decreased mean arterial pressure [3,4]. Additionally, a high dose of DMT used for sedation in pediatric patients causes hypertension, which is mediated by alpha-2 adrenoceptor stimulation of the vascular smooth muscles [5,6,7,8]. Lipid emulsion (LE) was originally developed for parenteral nutrition. Currently, LE is used not only for parenteral nutrition, but also for treatment of systemic toxicity of local anesthetic or non-local anesthetic drugs [9]. Additionally, DMT can be used to sedate patients receiving LE infusion for either parenteral nutrition or treatment of systemic toxicity caused by a toxic dose of local or non-local anesthetics. Increased fatty acids, which are derived from LE, enhanced blood pressure and systemic vascular resistance, and inhibited flow-mediated vasodilation, which seems to be caused by the decreased NO release of intact artery [10,11,12]. DMT-induced contraction of vascular smooth muscle is attenuated by endothelial nitric oxide (NO) release of intact aorta [13,14,15]. The phosphorylation of caveolin-1, which is upstream to the cellular signal pathway of NO production, induces endothelial nitric oxide synthase (eNOS) phosphorylation and NO release of intact vessel [16]. However, the detailed upstream cellular signal pathway associated with the DMT-induced NO release of intact artery remains unknown. LE inhibits acetylcholine-induced NO-mediated vasodilation [17]. Therefore, in terms of endothelial NO release, as LE attenuates NO-induced vasodilation and DMT-induced contraction is attenuated by endothelial NO release of intact aorta, LE may augment the DMT-induced contraction via inhibition of endothelial NO release of intact aorta [10,11,12,13,14,17]. The widely accepted underlying mechanism of LE treatment of local anesthetic systemic toxicity is lipid shuttling, wherein LE absorbs highly lipid-soluble drug (e.g., bupivacaine; log (octanol/water partition coefficient): 3.41) from vital organs, such as the heart, and subsequently, the LE containing the highly lipid-soluble drug is transported to the liver and adipose tissue for detoxification and storage [9,18]. As DMT is highly lipid-soluble (log (octanol/water partition coefficient) = 2.8: >2), LE may decrease the in vivo plasma DMT concentration via the absorption of DMT into the lipid phase of LE, leading to the altered hemodynamic response induced by DMT [19]. Considering the above stated factors (LE-mediated NO inhibition of intact artery and LE-mediated absorption of a highly lipid-soluble drug such as DMT), this exploratory study tested the biological hypothesis that LE has a biphasic effect on the vascular response induced by DMT, which is mainly dependent on the endothelium of intact artery. That is, LE may enhance the DMT-induced contraction of intact aorta with endothelium, while it may decrease the DMT-induced contraction of the endothelium-denuded aorta. However, the effect of LE on the DMT concentration and its induced contraction remains unknown. Therefore, this study aimed to examine the effect of LE (Intralipid) on the vasoconstriction induced by DMT in isolated rat aorta and to elucidate the associated cellular mechanism with a particular focus on endothelial NO release.

## 2. Results

**Figure 1:**
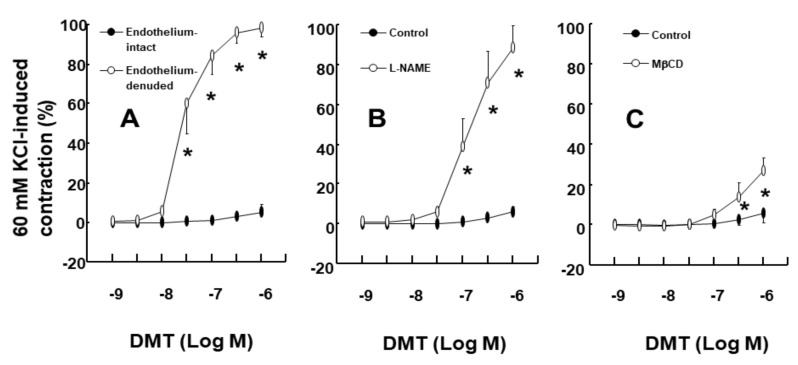
(**A**): Effect of endothelial denudation on the dexmedetomidine (DMT)-induced contraction in the isolated rat aorta. Data (*n* = 11) are shown as mean ± standard deviation and expressed as the percentage of isotonic 60 mM KCl-induced contraction. 60 mM KCl-induced contraction was 1.68 ± 0.30 and 2.22 ± 0.59 g for endothelium-intact and endothelium-denuded aorta, respectively. *n* indicates the number of isolated rat aorta. * *p* < 0.001 versus endothelium-intact aorta. (**B**): Effect of N^w^-nitro-L-arginine methyl ester (L-NAME, 10^−4^ M) on the DMT-induced contraction in the endothelium-intact rat aorta. Data (*n* = 6) are shown as mean ± standard deviation and expressed as the percentage of isotonic 60 mM KCl-induced contraction. 60 mM KCl-induced contraction was 1.83 ± 0.29 and 1.85 ± 0.35 g for control and L-NAME, respectively. *n* indicates the number of rats from which the thoracic aortae were obtained. * *p* < 0.001 versus control. (**C**): Effect of methyl-β-cyclodextrin (MβCD, 7 × 10^−3^ M) on the DMT-induced contraction in the endothelium-intact rat aorta. Data (*n* = 6) are shown as the mean ± standard deviation and expressed as the percentage of isotonic 60 mM KCl-induced contraction. 60 mM KCl-induced contraction was 1.81 ± 0.13 and 1.76 ± 0.27 g for control and MβCD, respectively. *n* indicates the number of rats from which the thoracic aortae were obtained. * *p* < 0.001 versus control.

**Figure 2:**
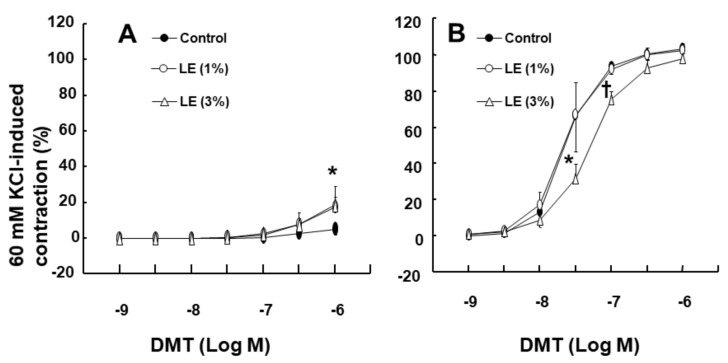
Effect of lipid emulsion (LE) on the dexmedetomidine (DMT)-induced contraction in the endothelium-intact (**A**, *n* = 6) and endothelium-denuded (**B**, *n* = 5) rat aortae. Data are shown as mean ± standard deviation and expressed as the percentage of isotonic 60 mM KCl-induced contraction. *n* indicates the number of rats from which the thoracic aortae were obtained. ^†^ *p* < 0.01 and * *p* < 0.001 versus control. (**A**): 60 mM KCl-induced contraction was 1.92 ± 0.04, 1.95 ± 0.14, and 1.97 ± 0.39 g for control, 1 and 3% LE, respectively. (**B**): 60 mM KCl-induced contraction was 1.77 ± 0.22, 2.02 ± 0.18, and 1.96 ± 0.12 g for control, 1 and 3% LE, respectively.

**Figure 3:**
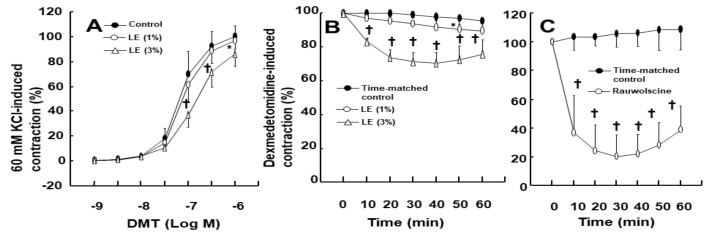
(**A**): Effect of lipid emulsion (LE) on the dexmedetomidine (DMT)-induced contraction in the endothelium-intact rat aorta pretreated with N^w^-nitro-L-arginine methyl ester (L-NAME, 10^−4^ M). Data (*n* = 6) are shown as mean ± standard deviation and expressed as the percentage of isotonic 60 mM KCl-induced contraction. 60 mM KCl-induced contraction was 1.78 ± 0.26, 1.85 ± 0.31, and 1.75 ± 0.11 g for control, 1 and 3% LE, respectively. *n* indicates the number of rats from which the thoracic aortae were obtained. * *p* < 0.05 and ^†^ *p* < 0.001 versus control. (**B**): Effect of LE on the DMT (10^−6^ M)-induced contraction in the endothelium-denuded rat aorta pretreated with L-NAME. Data (*n* = 8) are shown as mean ± standard deviation and expressed as the percentage of DMT (10^−6^ M)-induced contraction. DMT (10^−6^ M)-induced contraction was 1.61 ± 0.31, 2.12 ± 0.47, and 1.94 ± 0.27 g for time-matched control, 1 and 3% LE, respectively. *n* indicates the number of isolated rat aorta. * *p* < 0.05 and ^†^ *p* < 0.001 versus time-matched control. (**C**): Effect of rauwolscine (3 × 10^−6^ M) on the DMT (10^−6^ M)-induced contraction in the endothelium-denuded rat aorta. Data (*n* = 7) are shown as mean ± standard deviation and expressed as the percentage of DMT (10^−6^ M)-induced contraction. DMT (10^−6^ M)-induced contraction was 1.57 ± 0.43 and 1.71 ± 0.28 g for time-matched control and rauwolscine, respectively. *n* indicates the number of isolated rat aorta. ^†^ *p* < 0.001 versus time-matched control.

The DMT-induced contraction was much higher in the endothelium-denuded aorta than in the endothelium-intact aorta (Figure 1A; *p* < 0.001 at 3 × 10^−8^ M to 10^−6^ M). NOS inhibitor N^W^-nitro-L-arginine methyl ester (L-NAME, 10^−4^ M) greatly enhanced the DMT-induced contraction in the endothelium-intact aorta (Figure 1B; *p* < 0.001 vs. control at 10^−7^ M to 10^−6^ M). Cholesterol dissolving agent methyl-β-cyclodextrin (MβCD, 7 × 10^−3^ M), which leads to reduction of caveolin, slightly increased the DMT-induced contraction in the endothelium-intact aorta (Figure 1C; *p* < 0.001 vs. control at 3 × 10^−7^ M and 10^−6^ M). LE (1% and 3%) increased the DMT (10^−6^ M)-induced maximal contraction (Figure 2A; *p* < 0.001 vs. control) (standardized mean difference (SMD): 1% LE = 2.517; 95% confidence interval (CI): 1.00 to 4.03; 3% LE = 1.738; 95% CI: 0.41 to 3.07) in the endothelium-intact aorta. However, low concentration (1%) of LE had no effect on the DMT-induced contraction in the endothelium-denuded aorta, whereas high concentration (3%) of LE inhibited DMT-induced contraction (Figure 2B; *p* < 0.01 vs. control at 3 × 10^−8^ M and 10^−7^ M). Additionally, low concentration (1%) of LE did not significantly alter the DMT-induced contraction of the endothelium-intact aorta pretreated with L-NAME (10^−4^ M) (Figure 3A), whereas high concentration (3%) of LE inhibited the DMT-induced contraction (Figure 3A; *p* < 0.05 vs. control at 10^−7^ M to 10^−6^ M DMT). Furthermore, high concentration (3%) of LE inhibited the DMT (10^−6^ M)-induced maximal contraction in the endothelium-denuded aorta (Figure 3B; *p* < 0.001 vs. control in 10–60 min). Alpha-2 adrenoceptor inhibitor rauwolscine (3 × 10^−6^ M) inhibited DMT (10^−6^ M)-induced contraction in the endothelium-denuded aorta (Figure 3C; *p* < 0.001 vs. control in 10–60 min).

**Figure 4:**
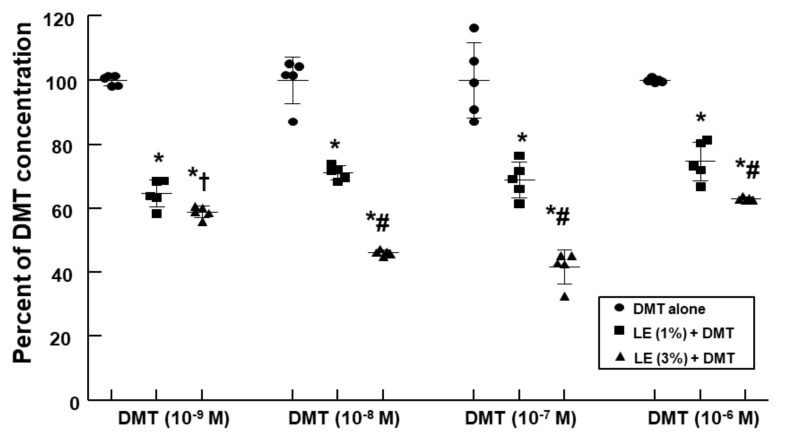
Effect of lipid emulsion (LE) on the dexmedetomidine (DMT) concentration in the Krebs solution. DMT concentration was analyzed using ultraperformance liquid chromatography-quadrupole time-of-flight mass spectrometry with multiple reaction monitoring mode. Data (*n* = 5) are shown as mean ± standard deviation and expressed as the percentage of each DMT (10^−9^, 10^−8^, 10^−7^, and 10^−6^ M) concentration alone. * *p* < 0.001 versus DMT alone. ^†^ *p* < 0.05 and ^#^ *p* < 0.001 versus LE (1%) + DMT.

LE (1% and 3%) decreased the DMT concentration (10^−9^, 10^−8^, 10^−7^, and 10^−6^ M) in a concentration-dependent manner (Figure 4; *p* < 0.001 vs. control; *p* < 0.05 vs. 1% LE plus DMT).

**Figure 5:**
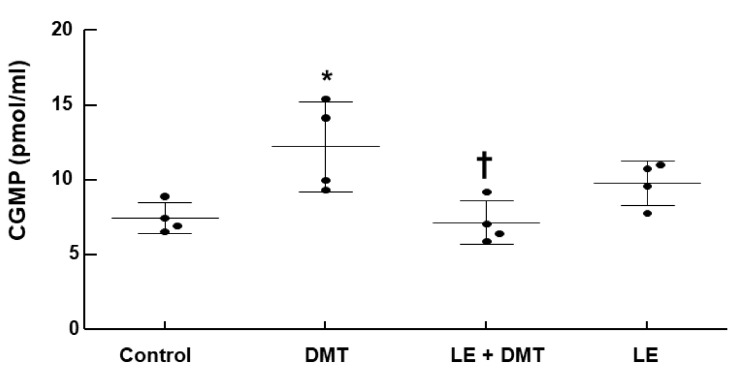
Effect of lipid emulsion (LE) on dexmedetomidine (DMT)-induced cyclic guanosine monophosphate (cGMP) formation in the isolated endothelium-intact rat aorta. Aortae were treated with DMT (10^−7^ M) for 5 min and with LE (1%) for 20 min, followed by treatment with DMT (10^−7^ M) for 5 min or LE (1%) alone for 25 min. Data (*n* = 4) are shown as mean ± standard deviation. *n* indicates the number of rats. * *p* < 0.05 versus control. ^†^ *p* < 0.001 versus DMT alone.

DMT (10^−7^ M) increased cyclic guanosine monophosphate (cGMP) formation in the endothelium-intact aorta (Figure 5; *p* < 0.05 vs. control), whereas LE (1%) decreased DMT (10^−7^ M)-induced cGMP production (Figure 5; *p* < 0.001 vs. DMT alone) (SMD: 1% LE = −2.132; 95% CI: −3.87 to −0.40).

**Figure 6:**
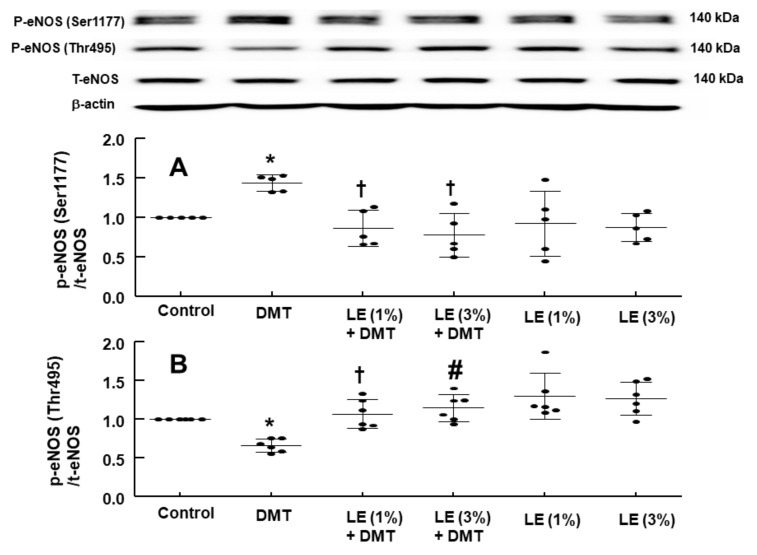
Effect of lipid emulsion (LE) and dexmedetomidine (DMT), alone or in combination, on stimulatory and inhibitory endothelial nitric oxide synthase (eNOS, Ser1177 (**A**) and Thr495 (**B**)) phosphorylation in human umbilical vein endothelial cells (HUVECs). HUVECs were treated with DMT (10^−6^ M) alone for 5 min and with LE (1% and 3%) for 1 h, followed by treatment with DMT (10^−6^ M) for 5 min or LE (1% and 3%) alone for 65 min. Data (*n* = 3) are shown as mean ± standard deviation. *n* indicates the number of independent experiments. * *p* < 0.05 versus control. ^†^ *p* < 0.01 and ^#^ *p* < 0.001 versus DMT alone. P-eNOS, phosphorylated eNOS; T-eNOS, total eNOS.

**Figure 7:**
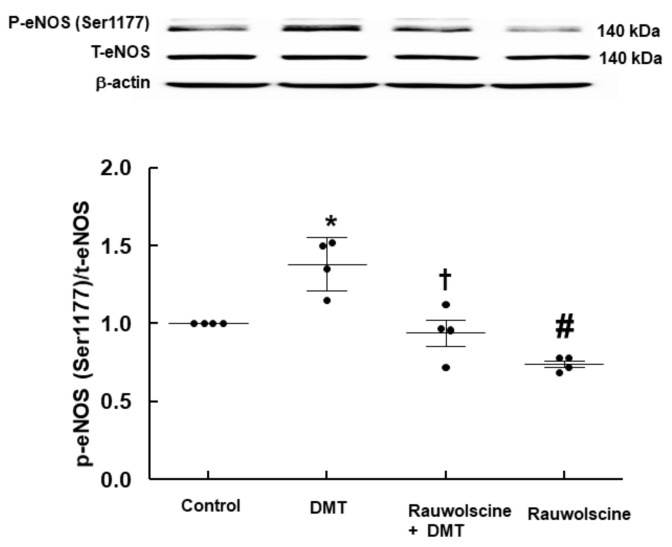
Effect of rauwolscine on dexmedetomidine (DMT)-induced endothelial nitric oxide synthase (eNOS, Ser1177) phosphorylation in human umbilical vein endothelial cells (HUVECs). HUVECs were treated with DMT (10^−6^ M) alone for 5 min and with rauwolscine (10^−6^ M) for 1 h, followed by treatment with DMT (10^−6^ M) for 5 min or rauwolscine (10^−6^ M) alone for 65 min. Data (*n* = 3) are shown as mean ± standard deviation. *n* indicates the number of independent experiments. * *p* < 0.01 and ^#^ *p* < 0.05 versus control. ^†^ *p* < 0.001 versus DMT alone. P-eNOS, phosphorylated eNOS; T-eNOS, total eNOS.

**Figure 8:**
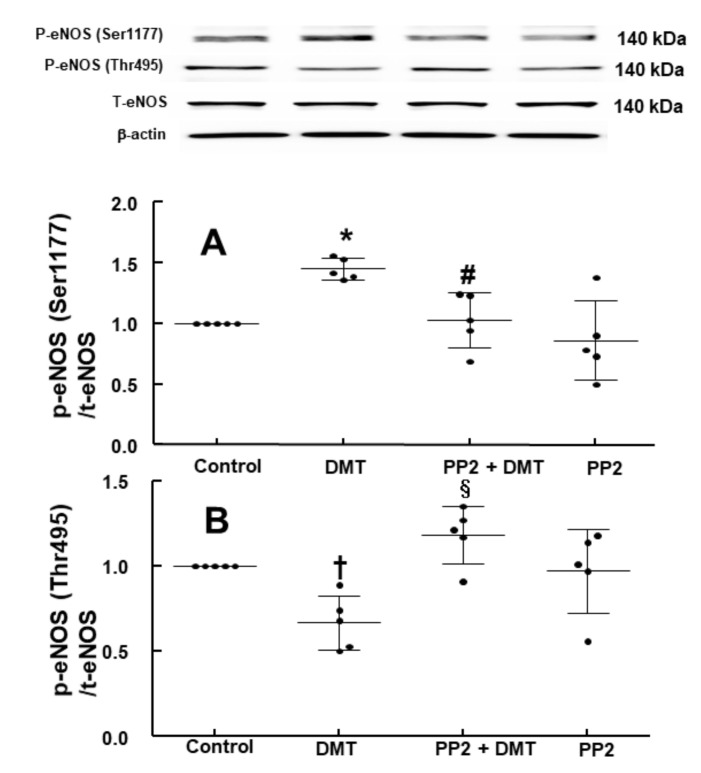
Effect of PP2 on dexmedetomidine (DMT)-induced stimulatory and inhibitory endothelial nitric oxide synthase (eNOS, Ser1177 (**A**) and Thr495 (**B**)) phosphorylation in human umbilical vein endothelial cells (HUVECs). HUVECs were treated with DMT (10^−6^ M) alone for 5 min and with PP2 (2 × 10^−5^ M) for 30 min, followed by treatment with DMT (10^−6^ M) for 5 min or PP2 (2 × 10^−5^ M) alone for 35 min. Data ((**A**): *n* = 5; (**B**): *n* = 4) are shown as mean ± standard deviation. *n* indicates the number of independent experiments. ^†^ *p* < 0.05 and * *p* < 0.01 versus control. ^#^ *p* < 0.05 and ^§^ *p* < 0.001 versus DMT alone. P-eNOS, phosphorylated eNOS; T-eNOS, total eNOS.

**Figure 9:**
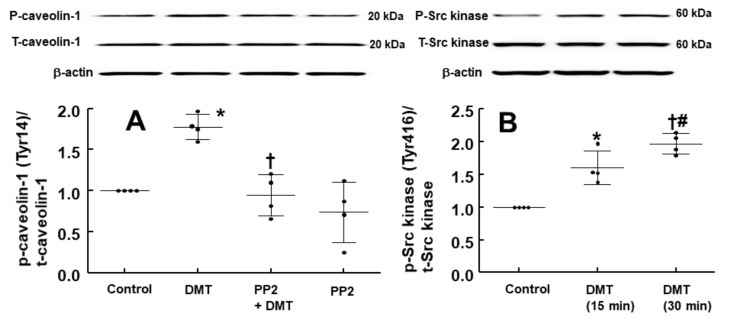
(**A**): Effect of PP2 on dexmedetomidine (DMT)-induced caveolin-1 (Tyr14) phosphorylation in human umbilical vein endothelial cells (HUVECs). HUVECs were treated with DMT (10^−6^ M) alone for 90 min and with PP2 (5 × 10^−6^ M) for 1 h, followed by treatment with DMT (10^−6^ M) for 90 min or PP2 (5 × 10^−6^ M) alone for 150 min. Data (*n* = 3) are shown as mean ± standard deviation. *n* indicates the number of independent experiments. * *p* < 0.01 versus control. ^†^ *p* < 0.001 DMT alone. P-caveolin-1, phosphorylated cavelin-1; T-caveolin-1, total caveolin-1. (**B**): Effect of DMT on Src kinase (Tyr416) phosphorylation in HUVECs. HUVECs were treated with DMT (10^−6^ M) alone for 15 min and 30 min. Data (*n* = 4) are shown as mean ± standard deviation. *n* indicates the number of independent experiments. * *p* < 0.01 and ^†^ *p* < 0.001 versus control. ^#^ *p* < 0.05 versus DMT (15 min). P-Src kinase, phosphorylated Src kinase; T-Src kinase, total Src kinase.

DMT (10^−6^ M) increased the stimulatory eNOS (Ser1177) phosphorylation (Figure 6A; *p* < 0.05 vs. control) in human umbilical vein endothelial cells (HUVECs), whereas LE (1% and 3%) inhibited DMT (10^−6^ M)-induced stimulatory eNOS (Ser1177) phosphorylation (Figure 6A: *p* < 0.01 vs. DMT alone). DMT (10^−6^ M) decreased inhibitory eNOS (Thr495) phosphorylation in HUVECs (Figure 6B; *p* < 0.05 vs. control), whereas LE (1% and 3%) reversed the decreased inhibitory eNOS (Thr495) phosphorylation induced by DMT (Figure 6B; *p* < 0.01 vs. DMT alone). The alpha-2 adrenoceptor inhibitor rauwolscine (10^−6^ M) decreased DMT (10^−6^ M)-induced stimulatory eNOS (Ser1177) phosphorylation (Figure 7; *p* < 0.001 vs. DMT alone) in HUVECs. Rauwolscine (10^−6^ M) alone inhibited eNOS (Ser1177) phosphorylation (Figure 7; *p* < 0.05 versus control). Src kinase inhibitor 4-Amino-3-(4-chlorophenyl)-1-(t-butyl)-1H-pyrazolo[3,4-d]pyrimidine,4-Amino-5-(4-chlorophenyl)-7-(t-butyl)pyrazolo[3,4-d]pyrimidine (PP2, 2 × 10^−5^ M) inhibited the increased stimulatory eNOS (Ser1177) phosphorylation induced by DMT (10^−6^ M) (Figure 8A; *p* < 0.05 vs. DMT alone) in HUVECs. However, PP2 (2 × 10^−5^ M) reversed DMT (10^−6^ M)-induced decreased inhibitory eNOS (Thr495) phosphorylation (Figure 8B; *p* < 0.001 vs. DMT alone). DMT (10^−6^ M) increased caveolin-1 (Tyr14) phosphorylation (Figure 9A; *p* < 0.01 vs. control), whereas PP2 (2 × 10^−5^ M) inhibited DMT (10^−6^ M)-induced caveolin-1 (Tyr14) phosphorylation (Figure 9A; *p* < 0.001 vs. DMT alone). DMT (10^−6^ M) induced Src kinase (Tyr416) phosphorylation in HUVECs (Figure 9B; *p* < 0.01 vs. control).

## 3. Discussion

**Figure 10:**
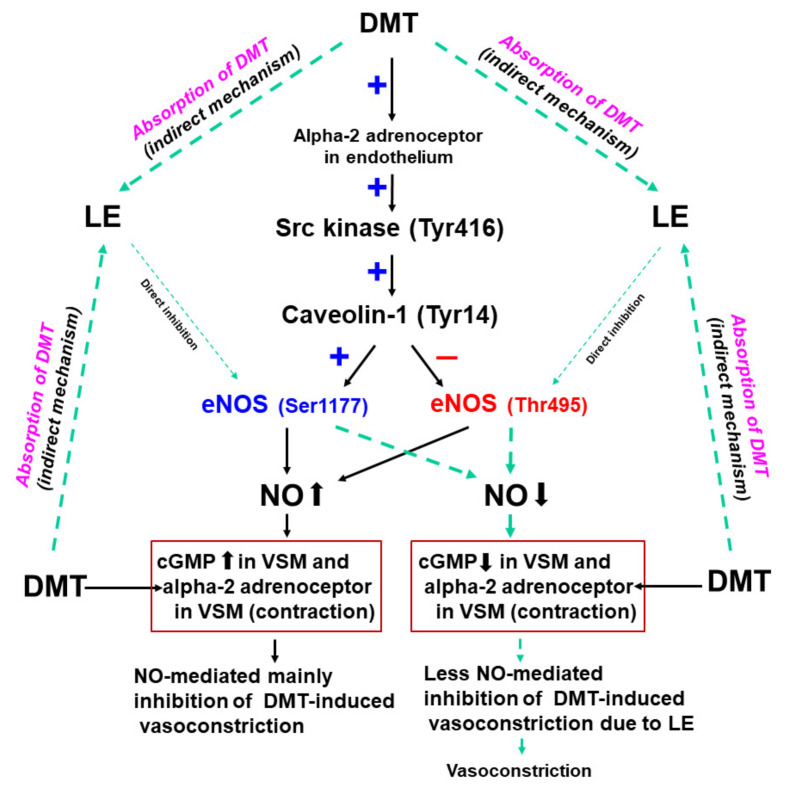
The putative cellular signal pathway associated with lipid emulsion (LE)-mediated enhancement of dexmedetomidine (DMT)-induced contraction of the rat aorta. As DMT-induced endothelial nitric oxide (NO)–mediated vasodilation of the aorta is prominently dominant compared with the DMT-induced vasoconstriction of the vascular smooth muscle (VSM), LE-mediated relatively less inhibition of the DMT-induced contraction leading to vasoconstriction, seems to be associated with the reduced amount of DMT available to endothelial alpha-2 adrenoceptor via absorption of DMT by LE. The DMT-induced NO production is due to the increased stimulatory endothelial nitric oxide synthase (eNOS Ser1177) phosphorylation and decreased inhibitory eNOS (Thr495) phosphorylation, which is mediated by the pathway involving alpha-2 adrenoceptor, Src kinase and caveolin-1. cGMP: cyclic guanosine monophosphate; +: stimulation, −: inhibition.

This is the first study suggesting that LE increases the DMT-induced contraction via the reversal of both stimulatory eNOS (Ser1177) and inhibitory eNOS phosphorylation (Thr495) induced by DMT, which seemed to be mediated by the pathway involving caveolin-1 and Src kinase (Figure 10). Our major findings are as follows: (1) NOS inhibitor L-NAME and MβCD increased the DMT-induced contraction in the endothelium-intact aorta; (2) LE (1%) increased the DMT-induced maximal contraction of the endothelium-intact aorta and decreased the DMT-induced cGMP formation; (3) LE decreased the DMT-induced stimulatory eNOS (Ser1177) phosphorylation, whereas it inhibited the DMT-induced attenuated inhibitory eNOS (Thr495) phosphorylation; and (4) LE decreased the DMT concentration.

Consistent with the findings of a previous report, the DMT-induced contraction was enhanced in the endothelium-denuded aorta than in the endothelium-intact aorta [13]. In agreement with the previous report’s findings, L-NAME and MβCD augmented the DMT-induced contraction of the endothelium-intact aorta (Figure 1B,C), suggesting that the DMT-induced contraction was attenuated by the pathway involving caveolin and NOS [13,14]. It was reported that LE decreases acetylcholine-induced NO-mediated vasodilation and increases left ventricular systolic pressure via inhibition of NO synthesis [17,20]. Additionally, increased non-esterified fatty acids released from LE enhance blood pressure and reduces flow-mediated vasodilation, which seems to be associated with the inhibition of NO synthesis [10,11,12]. Similar to the findings of previous reports, LE (1%) enhanced the DMT-induced maximal contraction of the endothelium-intact aorta (Figure 2A), whereas L-NAME pretreatment abolished LE (1%)-mediated enhancement of the DMT-induced contraction of the endothelium-intact aorta (Figure 3A), suggesting that LE (1%)-mediated enhancement of the DMT-induced contraction was caused by inhibition of NO synthesis, which seemed to be associated with the decreased concentration of DMT caused by LE [17,20]. As LE alone reduces NO synthesis, the complete separation of indirect (reduction of DMT concentration) and direct effect (direct inhibition of DMT-induced eNOS phosphorylation) of LE contributing to the inhibition of NO synthesis induced by DMT, was very difficult to achieve in our study (Figure 10) [17,21]. The high concentration of LE (3%) decreased DMT-induced contraction of the endothelium-denuded aorta and endothelium-intact aorta pretreated with L-NAME (Figure 2B and Figure 3A,B). LE was reportedly effective in alleviating the cardiovascular collapse induced by a toxic dose of non-local anesthetic drugs with high lipid solubility (log (octanol/water partition coefficient): >2), including amitriptyline, quetiapine, verapamil, amlodipine, and diphenhydramine, which appears to be associated with the absorption of the highly lipid-soluble drug from vital organs, such as the heart, into the lipid phase of LE, followed by enhanced redistribution [9,22,23,24]. Since DMT is highly lipid-soluble (log (octanol/water partition coefficient) = 2.8), a high concentration of LE (3%) produced a greater reduction of DMT concentration (from 10^−9^ M to 10^−6^ M) than a low concentration of LE (1%) (Figure 4). This effect seems to be related to the pharmacodynamic effect of LE on the DMT-induced contraction, which appears to be mainly due to less amount of DMT available to the endothelial alpha-2 adrenoceptor via the absorption of DMT by the lipid phase of LE. However, further study to examine the effect of LE on the hemodynamic response induced by DMT in an in vivo state is needed to determine whether this effect is due to pharmacodynamic or/and pharmacokinetic effects. This high concentration (3%) of LE-mediated inhibition of the DMT-induced contraction (Figure 2B and Figure 3A,B) in the endothelium-intact aorta pretreated with L-NAME or endothelium-denuded aorta seems to be caused by the greater absorption of DMT into the lipid phase of the high-concentration LE from the aorta [9,19,22,23,24]. The high concentration (3%) of LE increased the DMT-induced contraction in the endothelium-intact aorta (Figure 2A), whereas it decreased the DMT-induced contraction in the endothelium-denuded aorta (Figure 2B). We suppose that the magnitude of LE (3%)-mediated inhibition of NO release in the endothelium-intact aorta, which contributes to enhanced contraction, exceeds the magnitude of LE (3%)-mediated reduction of the DMT concentration in the vascular smooth muscles, which contributes to decreased contraction. However, a further study regarding the detailed mechanism associated with high concentration (3%)-mediated enhancement of the DMT-induced contraction of the endothelium-intact aorta is needed.

Caveolin-1 interacts with the upstream signaling molecule non-receptor tyrosine kinase and downstream signaling molecule eNOS through scaffolding domain, which leads to NO release [16,25]. The binding of caeveolin-1 and eNOS leads to the inhibition of eNOS and NO synthesis, whereas enhanced caveolin-1 phosphorylation increases eNOS phosphorylation and NO synthesis [16,25,26]. The phosphorylation of eNOS at Ser1177 increases NO synthesis, whereas the phosphorylation of eNOS at Thr495, which is a negative regulatory site, reduces NO synthesis [25]. Consistent with our finding of isometric tension measurement, DMT increased stimulatory eNOS (Ser1177) phosphorylation and decreased inhibitory eNOS (Thr495) phosphorylation, which leads to an increased NO synthesis. Additionally, similar to the LE-mediated enhancement of the DMT-induced contraction in the endothelium-intact aorta (Figure 2A and Figure 3A) via inhibition of NO synthesis, LE reverses both the increased stimulatory eNOS (Ser1177) phosphorylation and the decreased inhibitory eNOS (Thr495) phosphorylation induced by DMT (Figure 6A,B), leading to decreased NO release and cGMP formation (Figure 5). This effect seems to be mainly associated with the absorption of DMT by LE and partially caused by direct inhibition of NO release (Figure 10) because LE alone decreases cGMP formation [21]. Src kinase inhibitor PP2 attenuated the increased stimulatory eNOS (Ser1177) phosphorylation and the decreased inhibitory eNOS (Thr495) phosphorylation induced by DMT (Figure 8A,B), suggesting that DMT-induced eNOS (Ser1177 and Thr495) phosphorylation is mediated by Src kinase. Similar to the findings of previous reports, PP2 inhibited DMT-induced caveolin-1 phosphorylation (Figure 9A) [16,27]. Furthermore, DMT increased the Src kinase (Tyr416) phosphorylation (Figure 9B). Moreover, alpha-2 adrenoceptor inhibitor rauwolscine inhibited the DMT-induced eNOS phosphorylation and DMT-induced contraction (Figure 3C). In addition, rauwolscine-induced inhibition of eNOS (ser1177) phosphorylation (Figure 7) seems to be due to inverse agonism [28]. Altogether, these results suggest that an increased stimulatory eNOS (Ser1177) phosphorylation and reduced inhibitory eNOS (Thr495) phosphorylation by DMT are mediated by the pathway involving caveolin-1 and Src kinase via alpha-2 adrenoceptor (Figure 10) [16,25,26,27].

Our study suggests that the accidental administration of supraclinical dose of DMT (10^−6^ M), which is higher than the clinically relevant concentration of DMT (2 × 10^−9^–8 × 10^−9^ M) for sedation, may exaggerate severe vasoconstriction and hypertension in patients receiving LE infusion either to maintain 1% plasma triglyceride concentration suggested for the treatment of drug toxicity or for parenteral nutrition compared with patients not receiving LE [5,29]. Moreover, as the concentration of LE (1%) suggested for drug toxicity treatment decreases the DMT concentration (10^−9^ M and 10^−8^ M), which is approximately equivalent to the plasma concentration of DMT used for sedation, the dosage of DMT used for sedation should be increased in patients receiving LE infusion [5,29]. Concomitant administration of high-dose DMT and LE may cause less magnitude of vasoconstriction in patients with compromised endothelial function, leading to less degree of hypertension. Our study had some limitations. First, isometric tension measurement had used isolated rat aorta, whereas the study regarding phosphorylation of eNOS, caveolin-1, and Src kinase induced by DMT used HUVECs. Second, organ blood flow and peripheral vascular resistance are mainly determined by small resistance arterioles. However, we used the aorta, which is regarded as a conduit vessel [30]. Further studies using small resistance arterioles and arterial endothelium are warranted.

## 4. Materials and Methods

The experimental protocols (GNU-190207-R0007; 1 February 2019) were approved by the Institutional Animal Care and Use Committee of Gyeongsang National University. All experimental procedures were performed following the Guideline of the Care and Use of Laboratory Animal of National Institute of Health.

### 4.1. Preparation of the Isolated Aorta Obtained from Rats and Isometric Tension Measurement

Male Sprague-Dawley rats weighing 220–300 g (Koatech, Pyeongtaek, Gyeonggi-do, Korea) were sacrificed using 100% CO_2_ supplied to the cage via a small hole. The isolated rat aortae to be obtained from rats were prepared to measure the isometric tension as described previously [31]. The thorax of the rats was exposed, and the descending thoracic aortae were removed from the thoracic cavity. The removed aorta was immersed into Krebs solution contained in a Petri dish. The Krebs solution was composed of the following components: sodium chloride (118 mM), sodium bicarbonate (25 mM), glucose (11 mM), potassium chloride (4.7 mM), calcium chloride (2.4 mM), magnesium sulfate (1.2 mM), and monopotassium phosphate (1.2 mM). The connective tissue and fat surrounding the isolated aorta were removed under a microscope (Carl Zeiss™ Stemi™ DV4 Series Stereomicroscopes with LED Illumination, Carl Zeiss AG, Oberkochen, Germany). The isolated aorta was cut into 2.5 mm length segments. The endothelium of some isolated aorta was removed by inserting two 25-G needles into the aortic lumen and rolling the isolated aortic rings forward and backward. The isolated endothelium-intact and endothelium-denuded aortae were suspended in a Grass isometric transducer (FT-03, Grass Instrument, Quincy, MA, USA) attached to an organ bath maintained at 37 °C. Based on a previous study, 24.5 mN baseline resting tension was maintained for 90 min to reach equilibrium [32]. While the baseline resting tension was maintained, the existing Krebs solution was exchanged with fresh Krebs solution every 30 min. The pH of Krebs solution was maintained at 7.4 by aerating the Krebs solution with a gas containing 95% O_2_ and 5% CO_2_. The intact endothelium of the isolated thoracic aorta was verified using the following procedure: After phenylephrine (10^−7^ M) induced a sustained and stable contraction, acetylcholine (10^−5^ M) was added into the organ bath. The aorta with >85% acetylcholine-induced vasodilation was regarded as the endothelium-intact aorta. The functional endothelial denudation was verified using the following procedure. After phenylephrine (10^−8^ M), which is lower concentration than phenylephrine used for the endothelium intact aorta due to endothelial denudation, induced a sustained and stable contraction, acetylcholine (10^−5^ M) was added into the organ bath. The aorta with <15% acetylcholine-induced vasodilation was regarded as the endothelium-denuded aorta. The aorta with or without endothelium showing acetylcholine-induced vasodilation was washed with fresh Krebs solution several times to recover baseline resting tension. After the baseline resting tension was recovered, the contraction was induced by isotonic 60 mM KCl and was used for the reference value to express contraction induced by DMT. The aorta showing isotonic 60 mM KCl-induced contraction was then washed with fresh Krebs solution to restore baseline resting tension. Then, experimental protocols were performed as described below.

### 4.2. Experimental Protocol

First, the effect of endothelial denudation, NOS inhibitor L-NAME, and MβCD on the DMT-induced contraction was examined in the isolated rat aorta. DMT (10^−9^–10^−6^ M) was cumulatively added into the organ bath to generate DMT concentration-response curves in the isolated endothelium-intact or endothelium-denuded aorta. After the endothelium-intact rat aorta was pretreated with L-NAME (10^−4^ M) or MβCD (7 × 10^−3^ M) for 20 min, DMT (10^−9^–10^−6^ M) was cumulatively added into the organ bath to generate DMT concentration-response curves in the presence or absence of L-NAME or MβCD.

Second, the effect of LE (Intralipid: 1% and 3%) on the DMT-induced contraction in the endothelium-intact or endothelium-denuded rat aorta was examined. After the isolated aorta with or without endothelium was pretreated with LE (1% and 3%) for 20 min, DMT (10^−9^–10^−6^ M) was cumulatively added to the organ bath to generate DMT concentration-response curves in the endothelium-intact and endothelium-denuded aortae in the presence or absence of LE.

Third, the effect of LE on the DMT-induced contraction in the endothelium-intact aorta pretreated with L-NAME was examined. After the endothelium-intact aorta was pretreated with L-NAME (10^−4^ M) alone for 35 min or L-NAME (10^−4^ M) for 15 min, followed by treatment with LE (1% and 3%) for 20 min, DMT (10^−9^–10^−6^ M) was cumulatively added to the organ bath to generate the DMT concentration-response curves in the endothelium-intact aorta pretreated with L-NAME in the presence or absence of LE. Additionally, the effect of LE and rauwolscine on maximal contraction induced by DMT (10^−6^ M) in the endothelium-denuded aorta was examined. After DMT (10^−6^ M)-induced maximal contraction reached to plateau, LE (1% and 3%) and rauwolscine (3 × 10^−6^ M) were added into the organ bath to monitor DMT (10^−6^ M)-induced contraction in the presence or absence of LE or rauwolscine for 60 min.

### 4.3. Measurement of DMT Concentrations in Krebs Solution

DMT (10^−9^, 10^−8^, 10^−7^, and 10^−6^ M) emulsified with LE (Intralipid: 1% and 3%) in Krebs solution was measured using ultra-performance liquid chromatography-quadrupole time-of-flight mass spectrometry (UPLC-Q-TOF MS: Water, Milford, MA, USA) [33]. To extract DMT from lipid emulsified samples, emulsified samples were centrifuged at 75,000× *g* for 40 min, and the aqueous layer containing DMT was injected into an Acquity UPLC BEH C18 column (100 × 2.1 mm, 1.7 μm; Waters) equilibrated with water/acetonitrile (99:1) (containing 0.1% formic acid). DMT eluted with a linear gradient (1%–100%) of acetonitrile (containing 0.1% formic acid) at a flow rate of 0.35 mL/min for 5 min was analyzed using Q-TOF MS with multiple reaction monitoring and positive electrospray ionization mode. The sampling and capillary core voltage was set at 30 V and 3 kV, respectively. Desolvation and source temperature were set at 100 °C and 400 °C, respectively, while the desolvation flow rate was 800 L/h. Eluted DMT was monitored by the precursor and product ions of m/z 409.14 and 294.08, respectively. LockSpray with lucine-enkephalin ((M + H) = 556.2771 Da) was used to ensure reproducibility and accuracy of all analyses. All mass data were collected and analyzed using UIFI 1.8.2 (Waters).

### 4.4. Measurement of cGMP in the Isolated Endothelium-Intact Aorta

cGMP measurement was performed as described previously [31]. Acetylation reagent contained in cGMP Complete Kit (Abcam, Cambridge Science Park, Cambridge, England) was used to measure cGMP. The descending endothelium-intact thoracic aorta was immersed in Krebs solution in a 10 mL organ bath maintained at 37 °C for 60 min. The aortic strips from the same endothelium-intact aorta were treated with DMT (10^−7^ M) for 5 min, LE (1%) alone for 25 min, or LE (1%) for 20 min, followed by treatment with DMT (10^−7^ M) for 5 min. Aortic strips were then frozen in liquid nitrogen and homogenized in 0.1 M HCl. Acidic supernatants were acetylated, and cGMP measurement was performed using ELISA with a cGMP Complete Kit. The concentration of cGMP in each strip was expressed as pmol/mL.

### 4.5. Cell Culture

HUVECs (C-003-5C, American Type Culture Collection, Manassas, VA, USA) were grown in the endothelial cell medium (ECM) (ScienCell, Carlsbad, CA, USA) supplemented with 1% endothelial cell growth supplement (Sciencell) and 20% fetal bovine serum (ScienCell), 100 units/mL penicillin, and 100 μg/mL streptomycin (ScienCell). All cells were cultured in a humidified incubator with 5% CO_2_ at 37 °C. For all experiments, HUVECs were used between the third and fifth passages and were starved in serum-free ECM for 4 h before drug treatment.

### 4.6. Western Blot

Western blot analysis was performed using our previously described method [31]. Briefly, cell lysates were prepared in radioimmunoprecipitation assay buffer (Cell Signaling Technology, Beverly, MA, USA) containing protease inhibitor cocktail (Thermo Fisher Scientific, Rockfield, IL, USA) and phosphatase inhibitor cocktail (Thermo Fisher Scientific). Insoluble fragments were removed by centrifugation at 20,000× *g* for 15 min at 4 °C. The protein concentration of the supernatant was determined using a bicinchoninic acid protein assay (Pierce, Rockford, IL, USA). Thermal denaturation was performed at 100 °C for 10 min. Denatured samples were loaded into 8% sodium dodecyl sulfate-polyacrylamide gel electrophoresis and then transferred to a polyvinylidene difluoride membrane (Millipore, Bedford, MA, USA). After blocking with 5% bovine serum albumin or 5% skim milk at room temperature (22–28 °C) for 1 h, the membranes were incubated with primary antibodies (anti-phospho-eNOS at Ser1177 (1:1000), anti-phospho-eNOS at Thr 495 (1:1000), anti-eNOS (1:1000), anti-phospho-caveolin-1 at Tyr14 (1:1000), anti-caveolin-1 (1:2000), anti-phospho-Src kinase at Tyr416 (1:1000), anti-Src kinase (1:1000), and anti-β actin (1:10,000)) at 4 °C overnight. Membranes were then washed in tris-buffed saline with 0.1% Tween 20 (TBST, pH 7.6) and incubated with horseradish peroxidase-conjugated anti-rabbit or anti-mouse IgG 1:5000 for 1 h at room temperature. Following TBST washes, protein bands were developed using Westernbright^TM^ ECL Western blotting detection kit (Advansta, Menlo Park, CA, USA), and images were captured with ChemiDoc^TM^ Touch Imaging System (Bio-Rad Laboratories Inc., Berkely, CA, USA). Band intensities were quantified using Image Lab^TM^ Software v.3.0 (Bio-Rad Laboratories, Inc., Hercules, CA, USA).

### 4.7. Materials

All chemicals were commercially available and had the highest purity. DMT was obtained from Orion Pharma (Turku, Finland). L-NAME, MβCD, PP2, rauwolscine, phenylephrine, and acetylcholine were obtained from Sigma Aldrich (St. Louis, MO, USA). Intralipid (20%), which is composed of linoleic acid (53%), oleic acid (24%), palmitic acid (11%), alpha-linolenic acid (8%), and stearic acid (4%), was obtained from Fresenius Kabi AB (Uppsala, Sweden). Anti-eNOS antibody (#610297) was obtained from BD Bioscience (Franklin, NJ, USA). Anti-phospho-eNOS (Ser1177) (#9571), anti-phospho-eNOS (Thr495) (#9574), anti-Src kinase (#2108), anti-phospho-Src kinase (Tyr416) (#2101), anti-caveolin-1 (#3238), and anti-phospho-caveolin-1 (Tyr14) (#3251) antibodies were obtained from Cell Signaling Technology (Beverly, MA, USA). All chemical concentrations were expressed as the final molar concentration.

### 4.8. Statistical Analyses

Data are presented as mean ± standard deviation. Contraction induced by DMT was expressed as the percentage of maximal contraction induced by isotonic 60 mM KCl. The contraction induced by DMT (10^−6^ M) for 60 min after adding of LE in the organ bath was expressed as the percentage of maximal contraction induced by DMT (10^−6^ M) before the addition of LE. The effect of endothelial denudation, inhibitors and LE, alone or combined, on the contraction induced by DMT was analyzed using two-way repeated-measures analysis of variance (ANOVA), followed by Bonferroni’s multiple comparison test using Prism 5.0 (Graphpad software, Inc., San Diego, CA, USA). The effect of LE on each DMT concentration and DMT-induced cGMP formation was analyzed using one-way ANOVA, followed by Bonferroni’s multiple comparison test. The effect of DMT, LE, rauwolscine, and PP2, alone or combined, on eNOS, caveolin-1, and Src kinase phosphorylation was analyzed using one-way ANOVA, followed by Bonferroni’s test. The SMD was estimated to analyze the effect of lipid emulsion on the contraction and cGMP concentration induced by DMT in endothelium-intact aorta. SMD was calculated using the mean difference (of contraction and cGMP formation induced by DMT in the absence or presence of LE) divided by pooled standard deviation [34]. In this exploratory study, with a sample size in isometric tension measurement, the measurement of DMT and cGMP concentration was calculated using resource equation method [35]. The sample size of each group in 2, 3, and 4 groups is estimated to be 6–11, 5–8, and 4–6, respectively [35]. The calculated *p* value should not be considered to be hypothesis-testing, but descriptive. *p* values < 0.05 were considered statistically significant.

## 5. Conclusions

In summary, taking into consideration that the endothelial NO-induced vasodilation by DMT is greatly dominant compared to the vasoconstriction induced by the DMT in the vascular smooth muscle, LE (1%) increased the DMT-induced maximal contraction of the endothelium-intact aorta via inhibition of NO synthesis, which seemed to be mainly associated with the DMT concentration reduced by LE. DMT-induced NO synthesis seems to be caused by increased stimulatory eNOS (Ser1177) phosphorylation and decreased inhibitory eNOS (Thr495) phosphorylation, which is mediated by Src kinase-induced caveolin-1 phosphorylation via alpha-2 adrenoceptor. The above-mentioned effect may be observed in patients with intact artery reflecting physiologic situation. However, in compromised endothelial function showing reduced NO release, such as diabetes and atherosclerosis, LE may attenuate contraction induced by DMT predominantly acting on the vascular smooth muscle.

## Data Availability

The data presented in this study are available on reasonable request from the corresponding author.
